# Book Review: Cardiovascular Diseases: Genetic Susceptibility, Environmental Factors, and Their Interaction

**DOI:** 10.3389/fphys.2018.01570

**Published:** 2018-11-06

**Authors:** Chidinma Adanna Okolo, Esther Udo Asamudo

**Affiliations:** Department of Physiology, School of Biomedical Sciences, University of Otago, Dunedin, New Zealand

**Keywords:** cardiovascular diseases, cardiovascular diseases–epidemiology, ecophysiology, genetics, coronary artery disease

## Introduction

This book highlights various cardiovascular diseases affecting humans. It takes into account how the genetic component of the body interacts with environmental cues to influence the functioning and integrity of the cardiovascular system. In this review, outstanding factors were highlighted and criticisms were made regarding some aspects of the book.

## Review

This text was organized into 10 chapters, with each chapter having different combinations of contribution authors. The first chapter discussed atherosclerosis as a cardiovascular disease broadly. The questions as to “when, where, how and why” atherosclerosis occurs, were raised and addressed. In addressing atherosclerosis, the authors left no stone unturned when discussing its pathogenesis as well as disease progression. The involvement of mechanosensors, immunomodulatory responses, as well as biochemical drive toward atherosclerotic plaque formation were discussed in-depth. In addition, the global epidemiology of cardiovascular diseases was extensively discussed. Knowing that migration could impact heavily on susceptibility to ischemic heart disease, was an opener. Intriguingly, when highlighting how different food sources and groups could increase or alleviate the risk of cardiovascular diseases onset and progression, we appreciated the fact that the authors did not out rightly condemn or recommend certain food groups. Considering that lots of controversies surround foods, and as a rule, portion size and control is generally recommended, rather than total abstinence.

We got drawn to the statement in chapter 2, stating that the underlying pathology of cardiovascular disease is atherosclerosis. In our opinion, it could have been better worded that, atherosclerosis is the major underlying pathology of cardiovascular disease. In writing, the norm is to first write the full terminology with the abbreviation bracketed, before proceeding to use the abbreviated term in text in the write-up. Toward the ending chapter, knowing that gene-environment interactions could be complimentary and agonistic as well was helpful. Also, elucidated was how genes interact with themselves.

The striking thing about this book was that, despite the fact that it was written by different authors, they adopted similar framework and flow when addressing different aspects of cardiovascular diseases. The genetic influence, therapeutic options as well as clinical implications of the discussed disease were elucidated.

This book is highly recommended for a wider audience, as well as to researchers particularly involved to cardiovascular research and teaching.

## Author contributions

CO and EA both read the book and together drew out criticism and generally summarized the book. CO did the initial typing, while EA did the proof reading and final editing.

### Conflict of interest statement

The authors declare that the research was conducted in the absence of any commercial or financial relationships that could be construed as a potential conflict of interest.

